# Design and Optimization of an Inductive-Stub-Coupled CSRR for Non-Invasive Glucose Sensing

**DOI:** 10.3390/s25247592

**Published:** 2025-12-14

**Authors:** Zaid A. Abdul Hassain, Malik J. Farhan, Taha A. Elwi, Iulia Andreea Mocanu

**Affiliations:** 1 Electrical Engineering Department, Mustansiriyah University, Baghdad 1004, Iraq; zaidasaad_79@uomustansiriyah.edu.iq (Z.A.A.H.); malik.jasim@uomustansiriyah.edu.iq (M.J.F.); 2 Department of Automation and Artificial Intelligence Engineering, College of Information Engineering, Al-Nahrain University, Baghdad 10001, Iraq; taelwi82@nahrainuniv.edu.iq; 3 Telecommunications Department, National University of Science and Technology Politehnica Bucharest, 060042 Bucharest, Romania

**Keywords:** CSRR, non-invasive glucose measurement, glucose levels, resonator

## Abstract

This paper presents a high-sensitivity microwave sensor based on a modified Complementary Split Ring Resonator (CSRR) architecture, integrated with inductive stubs, for non-invasive blood glucose monitoring. The proposed sensor is designed to enhance the electric field localization and coupling efficiency by introducing inductive elements that strengthen the perturbation effect caused by glucose concentration changes in the blood. Numerical simulations were conducted using a multilayer finger model to evaluate the sensor’s performance under various glucose levels ranging from 0 to 500 mg/dL. The modified sensor exhibits dual-resonance characteristics and outperforms the conventional CSRR in both frequency and amplitude sensitivity. At an optimized stub gap of 2 mm, which effectively minimizes the capacitive coupling effect of the transmission line and thereby improves the quality factor, the sensor achieves a frequency shift sensitivity of 0.086 MHz/mg/dL and an amplitude sensitivity of 0.02 dB/mg/dL, compared to 0.032 MHz/mg/dL and 0.0116 dB/mg/dL observed in the standard CSRR structure. This confirms a significant enhancement in sensing performance and field confinement due to the optimized inductive loading. These results represent significant enhancements of approximately 168% and 72%, respectively. With its compact design, increased sensitivity, and potential for wearable implementation, the proposed sensor offers a promising platform for continuous, real-time, and non-invasive glucose monitoring in biomedical applications.

## 1. Introduction

Diabetes is a chronic disorder that needs to be diagnosed early and treated appropriately, in order to lower risks and enhance patient health. Over the past century, unhealthy lifestyles, such as an unbalanced diet and a lack of physical activity, have been widely blamed for the increasing prevalence of diabetes. In addition to lifestyle-related factors, the worldwide increase in diabetes prevalence is also influenced by longer life expectancy, improved healthcare accessibility, and broader medical screening coverage that leads to a higher number of diagnosed cases.

The World Health Organization (WHO) reports that the percentage of adults with diabetes rose from 4.7% in 1980 to 8.5% in 2014 [1]. About 1.6 million fatalities in 2015 alone were directly related to diabetes. In order to prevent complications from diabetes, such as cardiovascular disease, renal damage, vision loss, an elevated risk of stroke, and damage to the lower limbs, it is imperative to build a thorough diabetes management strategy. People with diabetes must have their blood glucose levels checked every day. This procedure usually calls for multiple measurements throughout the day, requiring each test to be performed with a lancet. The majority of glucose meters use electro-chemical processes, which require applying a drop of blood onto a test strip. Regular intrusive monitoring poses possible health hazards, such as contaminating the testing apparatus [2]. The everyday hazards, discomfort, and annoyance connected with conventional testing might be reduced by a non-invasive approach. Researchers have studied a variety of non-invasive blood glucose monitoring methods for more than 40 years [3]. For example, research in [3,4] investigated the examination of urine and tears as possible non-invasive glucose monitoring techniques. Although these methods make it possible to measure blood glucose levels without intrusive procedures, blood has a far higher glucose concentration than saliva, perspiration, and tears [4]. Moreover, these techniques are not ideal for ongoing observation. The necessity for high accuracy, low cost, ease of use, small form, and resistance to physiological and temperature fluctuations, however, make developing an efficient non-invasive glucose sensor difficult. Since diabetic patients must check their blood glucose levels frequently, including before meals, two hours after meals, before bed, etc., continuous and non-invasive monitoring is a preferred option [1]. In this regard, microsensors are becoming increasingly popular because of their ability to offer a practical way to accomplish continuous, non-invasive glucose monitoring without interfering with the patient’s everyday routine. For instance, variations in the dielectric characteristics in [4] can be used to monitor the levels of glucose non-invasively; ref. [4] suggested a radio wave transmission-based glucose meter that operates at frequencies ranging from 5 GHz to 12 GHz. This technology’s primary limitation is that it necessitates the use of high frequencies in order to mitigate the impact of skin on measurement accuracy. However, the “Photoplethysmography” method, which measures infrared absorption, is the basis for another glucose testing concept. However, this type of sensor faces the challenge of needing an additional sensor to detect the heart rate [5]. Over the past two decades, there has been an increasing interest in wireless technologies and their various applications, including sensing blood glucose concentration using microwave or radio frequency (RF) technologies.

In addition, the desire to design medical diagnostic devices stems from the study of the interaction of electromagnetic waves with physiological tissues, which includes the analysis of the dielectric properties and the anomalies of tissues [6,7,8]. Since the dielectric material’s characteristics are what primarily govern how electromagnetic waves behave within it, they are one of the primary considerations in the construction of a microwave or radio frequency framework. The study of electromagnetic waves’ effects on the human body has been accelerated by the development of body-centric and portable wireless communication systems, as well as the potential for implanted sensors to track biological processes. Another study looked into using a dielectric resonator running at 1.68 GHz to identify changes in glucose by observing changes in resonance frequencies. Nevertheless, this kind of sensor’s detection accuracy was significantly less than the 5 mg/mL threshold needed for human measurements [9].

Resonant cavity setups were developed previously [10,11], designed to operate at frequencies between 2 and 3 GHz. Other studies used antenna sensors with different frequency ranges, between 1 and 2.5 GHz [12] and 5 GHz to 8.5 GHz [13]. In addition, open-ended waveguide structures have been used to study the effect of varying glucose levels on the dispersion coefficients (S-parameters) of waveguides up to 20 GHz [14]. In [14], a metamaterial-based microfluidic sensor was proposed for detecting glucose concentration in aqueous solutions. The proposed sensor uses a capacitor between the fingers in the resonator, which provides high sensitivity for testing insulating liquids. Ref. [15] summarizes the design of a Wideband Antenna-Sensor System employed for the dielectric characterization of liquids, with a specific application in monitoring glucose concentrations in aqueous solutions. The sensing mechanism relies on measuring the changes in the reflection coefficient S11 and resonant frequency resulting from the variations in the relative permittivity of the fluid interacting with the antenna’s near-field Ref. [16] describes the development of a patch antenna components with phantoms, intended for an antenna operating at 4.75 GHz. The study included the evaluation of a range of liquid phantoms, such as pig blood and physiological solutions, with glucose levels ranging from 150 to 550 mg/dL. The results showed a linear frequency change at 5 MHz, with no correlation between the readings and changes in temperature or sample volume. A particular optimization process was used to create microwave sensors that have a steady response independent of changes in tissues other than blood in [17]. Different sensor configurations with associated resolution metrics were taken into account in order to evaluate the suggested concept and design strategy. In order to distinguish between concentrations in the range of 100 to 300 mg/dL, numerical findings validate the potential of achieving a good trade-off between the sensitivity toward blood glucose levels and the measurement stability against undesirable phantom changes. For liquid samples, a dual frequency microwave split ring resonator is demonstrated in [18], while a comparable approach is suggested in [19], where a two resonant frequency dual-sensing is used. A sensor for measuring glucose concentration non-invasively is also shown. There are reports of microwave resonators in a variety of shapes in [20,21,22,23,24]. Additional intriguing configurations with high Q-factors that are based on split ring resonators may be found in [25,26].

Beyond the sensor-oriented studies, many works have explored the versatility of Complementary Split-Ring Resonator (CSRR) structures in various microwave and photonic applications, including metasurface and metamaterial design based on the Babinet principle [27], microstrip bandpass filters [28], polarization conversion and chirality-based structures [29], and compact UWB antennas [30], demonstrating the wide applicability and design flexibility of CSRRs across multiple domains.

In this work, we present the design and optimization of a miniaturized microwave resonator based on a circular CSRR structure for non-invasive blood glucose monitoring. The study begins by reproducing a conventional CSRR sensor and quantitatively assessing its performance limitations, particularly the reduced sensitivity resulting from the presence of the microstrip line coupling capacitance. To overcome this drawback, an improved sensor configuration is proposed by integrating an inductive stub that effectively suppresses coupling capacitance, thereby enhancing the sensor’s responsiveness to variations in the dielectric properties of biological media. Moreover, the proposed design enables two-port reflection-based measurement (|S_21_|), contributing to a more compact, low-cost, and wearable-compatible sensing solution.

## 2. Conventional CSRR Microwave Glucose Biosensor

**Figure 1:**
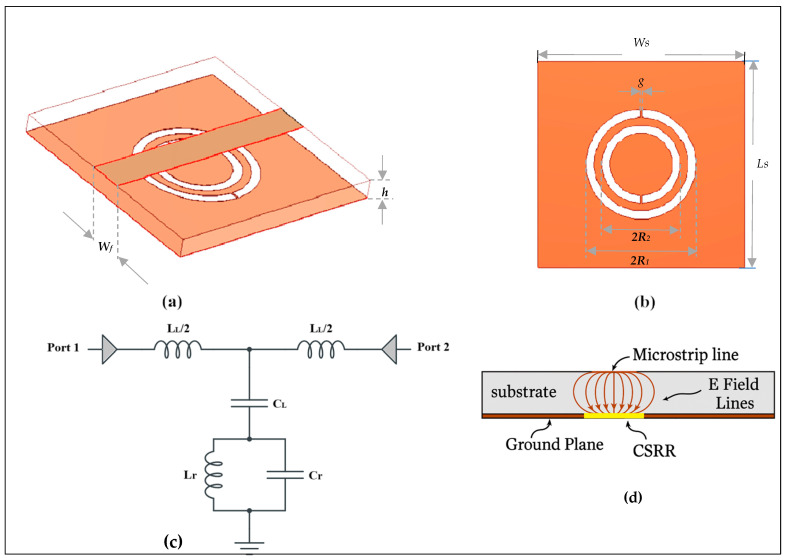
Topology of the basic CSRR unit cell: (**a**) 3D structure, (**b**) pop view, (**c**) equivalent lumped-elements circuit, (**d**) side view.

Figure 1 displays the unit cell of the CSRR loaded microstrip lines with CSRRs etched in the ground plane. The host microstrip line’s per-unit-cell inductance and capacitance are denoted by L_L_ and C_L_, while the CSRR total capacitance (*C_r_*) and inductance (*L_r_*) represent the electrical coupling on the ground. The structure’s physical attributes in Figure 1c are described by [31]:

(1) $$\varepsilon =\frac{Y_{p}}{jw}=\frac{Cc\left(1-w^{2}L_{r}C_{r}\right)}{1-w^{2}\left(C_{L}+C_{r}\right)L_{r}}$$

$$for\;\varepsilon <0:\;f_{s}<f<f_{o}$$

where ε is the complex dielectric constant, $f_{s}=\frac{1}{2\pi \sqrt{L_{R}\left(C_{L}+C_{r}\right)}}$, is the short circuit frequency that eliminates the parallel impedance, while, $f_{o}=\frac{1}{2\pi \sqrt{L_{r}C_{r}}}$, is the open circuit frequency that eliminates the parallel admittance. The CSRR unit cell depicted in Figure 1. was built and simulated at different values of the substrate lengths to emphasize the negative permittivity frequency range, which is provided by lower and upper frequencies (*f_s_*, *f_o_*) using FR4 substrate having ε_r_ = 4.4 and tanδ = 0.0035, with the physical dimensions given in Table 1.

**Figure 2:**
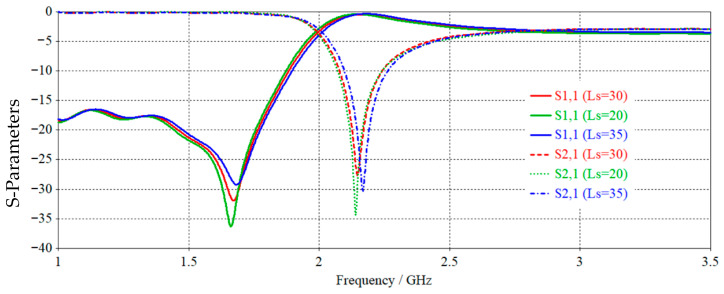
Simulated S-parameters (S_11_ and S_21_) of the unit cell for different stub lengths: Ls = 20, 30, and 35 mm.

According to Figure 2, the CSRRs have a resonance frequency at different values at unit cell length of about 2.16 GHz. In this instance, the negative permittivity band of the unit cell interval is 20.276% fractional bandwidth, which is constrained by lower and upper frequencies.

**Figure 3:**
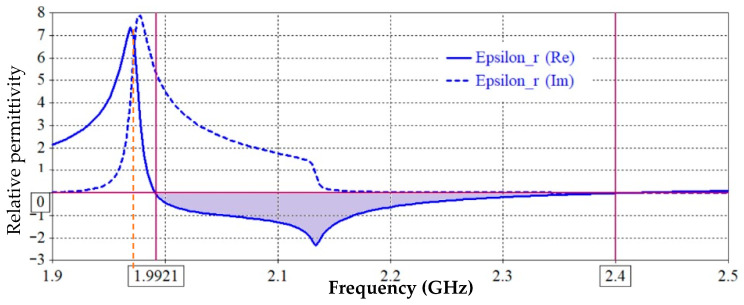
Extracted effective permittivity (ε_r_) response of the proposed unit cell showing both real and imaginary parts versus frequency, indicating the negative permittivity region near the resonance frequency.

The basic characteristics that are utilized to derive the equivalent circuit must be explained after the negative permittivity band and associated critical frequencies have been clarified. Figure 3 shows the Re ε and the Im ε, as well as the critical frequencies (*f_s_*, *f_o_*, as well as the center or resonance frequency *f_r_*). The resonance frequency that nullifies the parallel impedance (transmission zero frequency), which is located at Re ε < 0, can alternatively be referred to as the other characteristic frequency. As is demonstrated in the following section, the extraction process demonstrates how the computation of an analogous circuit directly depends on these three frequencies. Since the electrical dimensions of the CSRRs are modest at resonance, lumped-element equivalent circuits can be used to represent the unit cell. The method clarifies that the equivalent circuit model of a transmission line loaded with CSRR depends critically on the effect of the negative permittivity band. The host microstrip line’s inductance *Lc* and coupling capacitance Cc are calculated from the lumped circuit in Figure 1c as the host transmission line per-unit-cell inductance and capacitance [32]:

(2) $$L_{c}=\left\{\begin{array}{c} \frac{60l}{c}ln\left[\frac{8h}{wf}+\frac{w}{4h}\right]\;\;\;\;\;\;\;\;\;\;\;\;\;\;\;\;\;\;\;\;\;\;\;\;\;\;\;\;\;\;\;\;\;\;\;\;\;\;\;\;\;\;\;\;\;\;for\;\frac{wf}{h}\leq 1\\ \frac{120\pi l}{c}\left[\frac{1}{\frac{wf}{h}+1.393+0.667\ln\left(\frac{wf}{h}+1.444\right)}\right]\;\;\;\;\;for\;\frac{wf}{h}>1 \end{array},\right.$$

(3) $$C_{c}=\left\{\begin{array}{c} \frac{\varepsilon_{r}l}{60cln\left[\frac{8h}{wf}+\frac{wf}{4h}\right]}\;\;\;\;\;\;\;\;\;\;\;\;\;\;\;\;\;\;\;\;\;\;\;\;\;\;\;\;\;\;\;\;\;\;\;\;\;\;\;\;\;\;\;\;for\;\;\frac{wf}{h}\leq 1\\ \frac{\varepsilon_{r}l\left[\frac{wf}{h}+1.393+0.667\ln\left(\frac{wf}{h}+1.444\right)\right]}{120\pi c}\;\;for\;\frac{wf}{h}>1\; \end{array}\right.$$.

**Figure 4:**
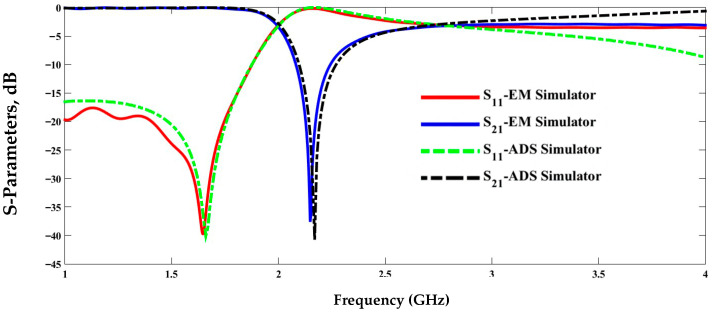
Magnitude of the transmission and reflection coefficient with respect to the frequency.

The resonance frequency *f_r_* can be directly identified from Figure 3, or using full wave electromagnetic simulation, or it can be extracted as denoting the midpoint of the negative permittivity band interval $\left(f_{r}=\frac{f_{0}+f_{s}}{2}\right)$ as shown in Figure 4 along with the frequency, located at Re ε < 0. This feature of the negative permittivity band is thought to be advantageous.

The higher frequency at which Re ε = 0 is used to directly determine the frequency *f_o_*, while the low frequency determines *f_s_*. Consequently, a reflection representation is not necessary as in the conventional method, and the Smith chart’s coefficient (S11) curve can be used to determine it [33]. This is the second benefit offered by the negative permittivity band features. The parallel impedance (*L_r_*, *C_r_*) of the composite can be found using *f_s_*, *f_o_*,. The process outlined above is utilized to acquire the electrical parameters of the CSRR unit cell, which is created and simulated in Figure 1c., and the results are synthesized in Table 2 to show the validity of the suggested technique.

Electrical simulation using the Advanced Design System (ADS) and electromagnetic modeling, as shown in Figure 4, can be used to obtain zero transmission in the frequency response of the structure from the elements in Table 2. The structure model and the evaluated lumped circuit extraction are validated by the good agreement between the electrical and electromagnetic simulations.

## 3. CSRRs Based Microwave Sensor Design

### 3.1. Proposed Sensor Configuration and Features

In CSRR-based configurations, the resonators are typically excited through electromagnetic coupling with the quasi-TEM mode of the guiding structure, such as a microstrip transmission line (MTL). This mode produces a high-intensity electric field concentrated near the dielectric interface, so that any CSRR element integrated within the ground plane, with a dielectric substrate mechanically supporting it, should be positioned within this strong-field region to ensure efficient excitation.

**Figure 5:**
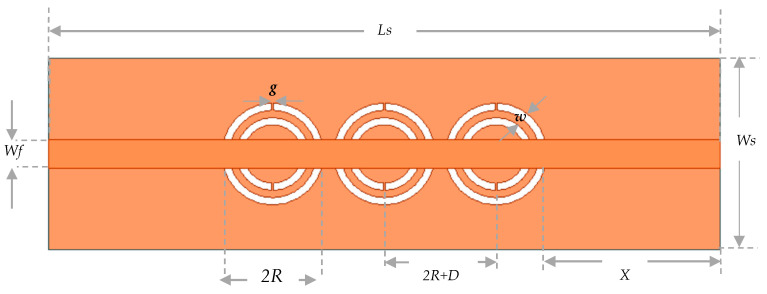
Top view of the metamaterial CSRR cells arranged in the dielectric substrate’s copper ground layer.

This spatial limitation restricts the sensing area and, consequently, the sensor’s sensitivity. To overcome this limitation, three CSRR cells are needed instead of one. When these cells are placed close to each other, mutual coupling occurs between them, resulting in a larger region of concentrated electric field. This helps to enhance the effective sensing volume of the structure. According to the numerical design, the suggested biosensor will function at about 2.3 GHz. In fact, using this frequency range would enable sufficient penetration depth for the produced electromagnetic waves to enter the fingertip blood arteries. The design of the proposed sensor is mainly based on the fundamental geometry of split-ring resonators with localized elements. On top of a dielectric substrate with dimensions of length *Ls*, width *Ws*, and thickness *h*, the sensor structure is made up of three identical cells of complementary split-ring passive resonators (CSRRs), arranged horizontally as shown in Figure 5. The initial cell is situated at a distance (X) away from the input port. As seen in Figure 5, the CSRR cells are spatially separated by D distance ranges between cell boundaries. A copper ground plane has been etched with two concentric split-rings of dielectric slits that are nestled inside one another to form each CSRR cell. As illustrated in Figure 4, the double split-rings are made to resemble two circular annular loops with a gap g between them. Every ring’s loop terminates in a g-wide metallic slot. Through a copper microstrip-line (MTL) with *L_f_* length and *W_f_* width etched on the bottom face of the dielectric substrate, the three resonators are connected to the RF power source. This sensor structure creates inductances and capacitances with stored oscillatory energies (both magnetic and electric) by inducing charges and currents inside the engraved dielectric rings. After both energies are matched, the microstrip sensor resonates at a particular frequency. The physical parameters of the sensor geometry and the dielectric specifications of the base substrate, in theory, dictate the intrinsic resonance properties (resonant frequency, width, depth, and Q-factor) [34].

Any permittivity mutations in the MUT can be linked to the generated effective capacitance of the resonant structure because the stored electric energy/field interacts with the surrounding media or MUT (i.e., fingerprint) put next to the CSRRs. This CSRR sensor’s glucose sensing mechanism is based on the capacitive disturbance brought on by altering the electric field distribution when the glucose sample is present. In order to obtain the maximum interaction with the coupled fields at the resonance frequency *f_r_*, this sample is applied to the CSRR surface at an area where the electric field is substantially concentrated. By carefully examining the altered resonance behavior, the ensuing variations in resonance profile are thought to be a hallmark of the sample’s electrical characteristics that might be connected to the corresponding glucose level.

The proposed structure is designed via the finite element based HFSS simulator, and its geometrical parameters are set appropriately to confine the coupled electric fields within the permittivity sensing region and reinforce the resonance strength. This results in a higher sensitivity that is sufficient for detecting slight variations in the dielectric properties of fingerprint fantom samples. The proposed sensor is a design using FR4 substrate (ε_r_ = 4.4 and tanδ = 0.02) with the following structure dimensions shown in Table 3.

**Figure 6:**
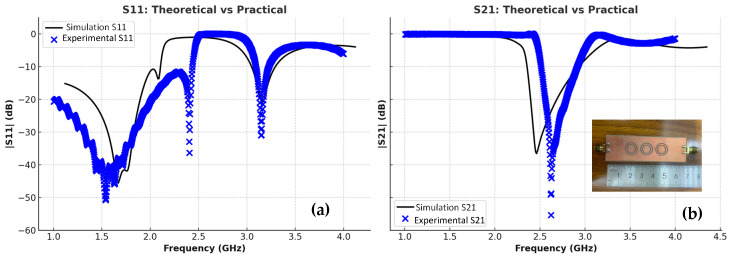
CSRR experimental and simulation: (**a**) |S11|, (**b**) |S21|.

The transmission and reflection scattering response (S21 and S11 coefficients) is first examined numerically in the unloading stage, as shown in Figure 6, with the resonance frequency around 2.48 GHz, with a wide –3 dB bandwidth and a steeper resonance depth of roughly –61 dB. Figure 6 presents a comparative analysis between the simulated (theoretical) results shown by the black lines and the experimentally measured results represented by the blue markers for both reflection S_11_ and transmission S_21_ parameters. As illustrated in the left plot, the measured S_11_ response closely follows the simulated trend, with minor deviations in resonance depth and frequency that can be attributed to fabrication tolerances, measurement uncertainties, and environmental influences. Similarly, the right plot shows a good agreement in the S_21_ behavior, with slight differences in attenuation levels and bandwidth. Overall, the close correspondence between simulation and measurement validates the accuracy of the proposed sensor model and confirms its reliable performance under practical experimental conditions. In the frequency range of 2.0–2.5 GHz, both the simulated and experimental results show a good agreement, with nearly identical resonance dips in |S11| and |S21|, confirming that the sensor maintains stable and consistent performance before phantom loading.

### 3.2. Finger Phantom Modeling and Glucose Concentration Assessment

**Figure 7:**
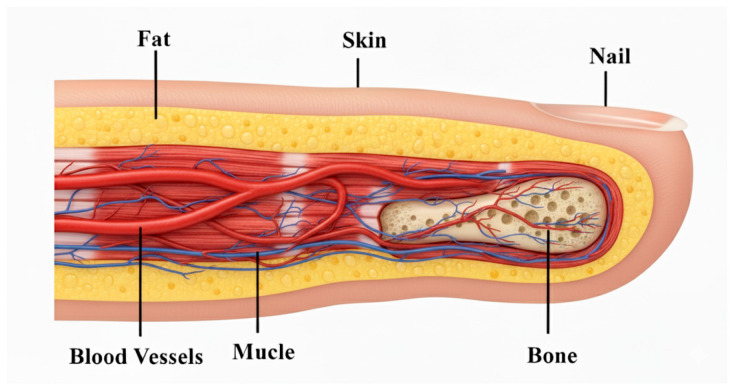
Cross-sectional anatomy of the index finger illustrating skin, fat, muscle, blood vessels, bone, and nail.

To make the numerical analysis easier, it is appropriate to build the phantom by striking a balance between the physical target description and the complexity. This is accomplished by creating a finger phantom model in an HFSS environment that simulates the actual human finger as shown in Figure 7. Dielectric materials with varied dielectric constants and conductivities are used to simulate the different layers of the finger, including the skin, fat, muscle, blood, and bone. The thicknesses of the different layers of the finger phantom, as well as their relative dielectric constants, are listed in Table 4 [17,35]. It has been noted that blood cell electrical characteristics are influenced by glucose content. Conductivity, relative permittivity, loss tangent, and other characteristics are examples of the electrical qualities. The dielectric constant, rather than glucose concentration in mg/dL, g/mL, or mol/L, can also be used to explain glucose concentration. The Cole–Cole model describes the frequency-dependent electrical behavior of blood cell glucose concentration. The most popular technique for figuring out biological tissues’ precise and efficient dielectric characteristics across a broad frequency range is the Cole–Cole model. Equations define the relationship between relative permittivity and conductivity at a given resonance frequency as follows:

(4) $$\hat{\varepsilon }\left(\omega \right)=\varepsilon_{\infty }+\sum_{n}\frac{\Delta \varepsilon_{n}}{1+{\left(j\omega \tau_{n}\right)}^{1-\alpha_{n}}}+\frac{\sigma_{i}}{j\omega \varepsilon_{o}}$$

where $\hat{\varepsilon }(\omega )$ is the complex permittivity as a function of angular frequency ω, ε_∞_ is the permittivity at very high frequencies, Δε_n_ is the permittivity difference for each relaxation process, τ_n_ is the relaxation time for each process, α_n_ is the fractional order parameter representing non-ideal relaxation behavior for process n, and σ_I_ is the ionic conductivity.

### 3.3. Numerical Evaluation

This section examines the functionality of the proposed loaded sensor, specifically its resilience to changes in glucose variation. In view of this, we take on phantom with a few features given in the previous section. The outcomes are displayed by calculating the previously mentioned metrics and displaying the behaviors of the scattering parameters. The finger phantom is placed above the ground plain at a distance of 1 mm. To illustrate, the behavior of the glucose sensing, the capacitor element C_m_ in Figure 4 acts as a variable capacitor due to the glucose variation, then the resonance frequency, with a change from some reference value (here the zero glucose level), is based on the fundamental parallel resonance formula:

(5) $$f_{r}≅\frac{1}{2\pi \sqrt{L_{R}\left(C_{c}+C_{R+}C_{M}\right)}}$$

For this case, the change in the reflection coefficient magnitude and frequency shift is studied by varying the concentration of glucose levels from 0 to 500 mg/dL in the finger phantom capillaries. The fluctuating glucose spectrum of diabetics (0–500 mg/dL) causes relatively small changes in the EM dispersive characteristics of blood tissues (ε_r_ and tanδ). In order to maximize the resonance strength and precisely confine the resonating electric fields within the permittivity sensing region, we must optimize the design of the suggested sensing structure for blood glucose monitoring applications with respect to the geometric parameters defining the planar MTL and the CSRR resonating cells. Obtaining greater sensitivity that can detect the slight variation in blood glucose dielectric characteristics is necessary. The selection of combining three cells and other design parameters is examined in the sections that follow, after careful examination of the sensitivity performance at various CSRR cell counts, resonator orders (i.e., single or double loop), excitation schemes, substrate thickness, and CSRR geometries. For these numerical analyses and modeling, the HFSS numerical simulator, which is based on the Finite Element Method (FEM), is used. A portion of the loaded glucose sample and the dielectric substrate is polarized by the plugged electric field in the MTL sensor structure when it is connected with the CSRRs. Increasing the dependence of the lowest transmission resonant frequency on the loaded sample permittivity (i.e., C_M_) could improve the sensor’s sensitivity to changes in glucose levels. The polarized fraction of the loaded sample is increased, while the dielectric characteristics (ε_r_ and tanδ) and thickness (h) of the substrate are decreased to decrease the field confinement inside the substrate. The resonant properties of the TP-CSRR sensor (*f_r_* and Q) depend on the geometrical parameters of each CSRR cell (diameters of the inner and outer rings a, coupling (g), and ring widths), in addition to the substrate specifications. The main impact of each of these parameters on *f_R_* was examined using HFSS.

**Figure 8:**
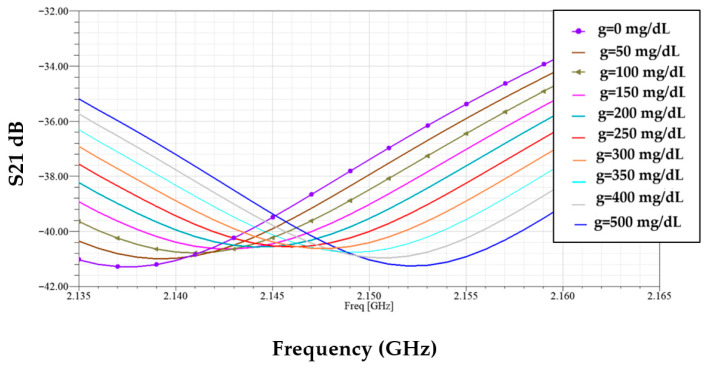
Transmission coefficient responses of various glucose samples.

**Figure 9:**
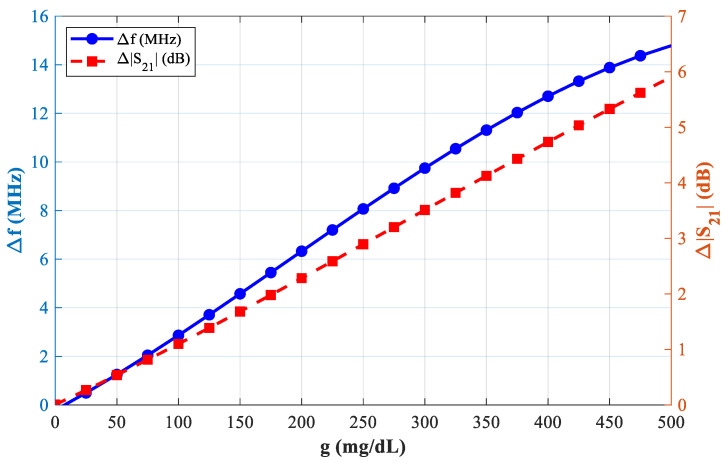
Sensor performance: ∆f, and ∆S_21_.

Figure 8 demonstrates the simulated transmission responses (S21) for glucose level samples between 0 mg/dL to 500 mg/dL, each sample loaded in the blood layer of the phantom. The enclosed plot shows these resonant amplitude (min of |S21|) variations at different glucose levels, which are given in Figure 9.

The average sensitivity of the sensor, in particular, can be approximated as follows:

(6) $$Sensitivity=\frac{{∆f}_{max}-{∆f}_{min}}{g_{max}-g_{min}}$$

This could be used to gauge how sensitive the suggested sensor is to the amount of glucose present. Our calculated slope is roughly 0.032 MHz/(mg/dL) and 0.0116 dB/mg/dL.

## 4. Coupling Theory and Sensitivity Enhancement

**Figure 10:**
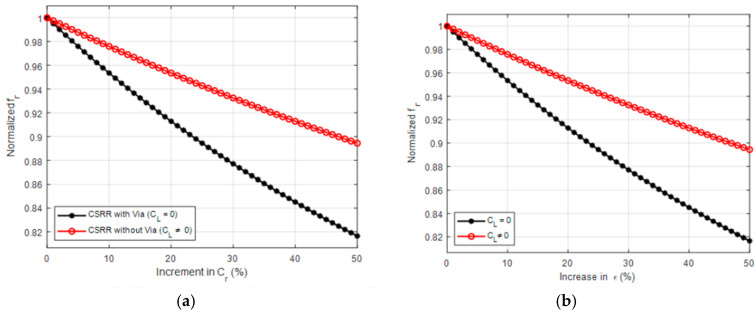
Normalized frequency variation with (**a**) *C_L_*, (**b**) ε.

Defected-ground resonators, like CSRRs, can be excited by means of the microstrip line, which is perpendicular to the resonators’ surface, making them suitable for use as near-field sensors. Figure 1a exhibits the sensor’s cross-section coupled with the quasi-TEM-TLs estimated electric field lines. In Figure 1, the normal electric field component can be represented as a parallel capacitance (*C_L_*) between the ground plane and power plane, while the TL’s magnetic field is represented as an inductance (*L_L_*). A potential difference between the ground plane and the resonator’s central island will be produced by the electric field which is represented as the resonator’s capacitance (*C_r_*). A circulating surface current, represented as an inductance (L_r_), will be produced on both sides of the resonator (the substrates and the empty space) by the potential difference. This makes the resonator suitable for designing near-field sensors. According to [36], the sensitivity of CSRRs is dependent on *C_r_*, hence reducing the sensor’s reliance on *C_L_* (e.g., *C_L_* = 0) would increase sensitivity. Figure 10 illustrates the relationship between normalized frequency (*f_r_*) and the variation of *Cr* (Figure 10a) as well as the variation in ε (Figure 10b) in two different conditions; one where C_L_ is present and another where it is absent. The X-axis represents the percentage increase in (*C_r_*), ranging from 0% to 50%, while the Y-axis shows the normalized frequency (*fr*), which varies between 0.82 and 1. Observing the condition of *C_L_* = 0, it is evident that as (*Cr*) increases, the normalized frequency drops sharply, indicating that the system is highly sensitive to *Cr* changes.

**Figure 11:**
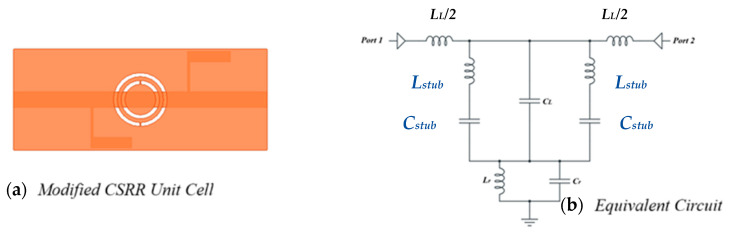
Modified CSRR: (**a**) unit cell geometry, and (**b**) its equivalent circuit model; *C_L_* represents the transmission line coupling capacitance.

Therefore, it can be said that reducing the CSRR-based sensors’ reliance on the TL capacitive coupling will improve both the coupling and sensitivity at the same time. Other coupling types, such as reactive load coupling, can be taken into consideration in order to study this. An inductor (*L_stub_*) and capacitor (*C_stub_*) parallel to *C_L_* can be included by re-examining the circuit model of the CSRR in Figure 1. It is possible to forecast that the system will display a band stop response based on the values of the shunt circuit tank using the modified circuit model. Figure 11 displays the proposed sensor and the expected circuit model, respectively. Therefore, it is anticipated that the sensitivity and coupling factor of the CSRR would be improved and that it will be possible to use it as a dielectric sensor based on the research in references and the proposed new coupling method.

**Figure 12:**
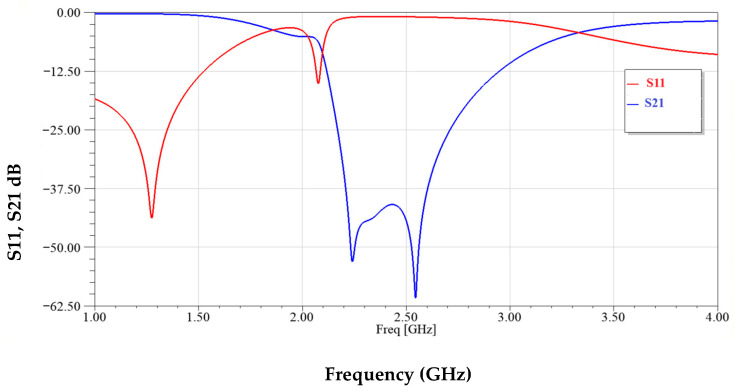
Modified CSRRs structure simulated transmission and reflection coefficients.

Figure 12 illustrates the frequency response of the sensor’s reflection coefficient (S_11_) and transmission coefficient (S21) over the range of 1–4 GHz. Two distinct resonance dips are observed in S21 around 2.3 GHz and 2.5 GHz, indicating dual-mode operation. These deep notches demonstrate strong electromagnetic coupling and high sensitivity to variations in the surrounding dielectric environment.

**Figure 13:**
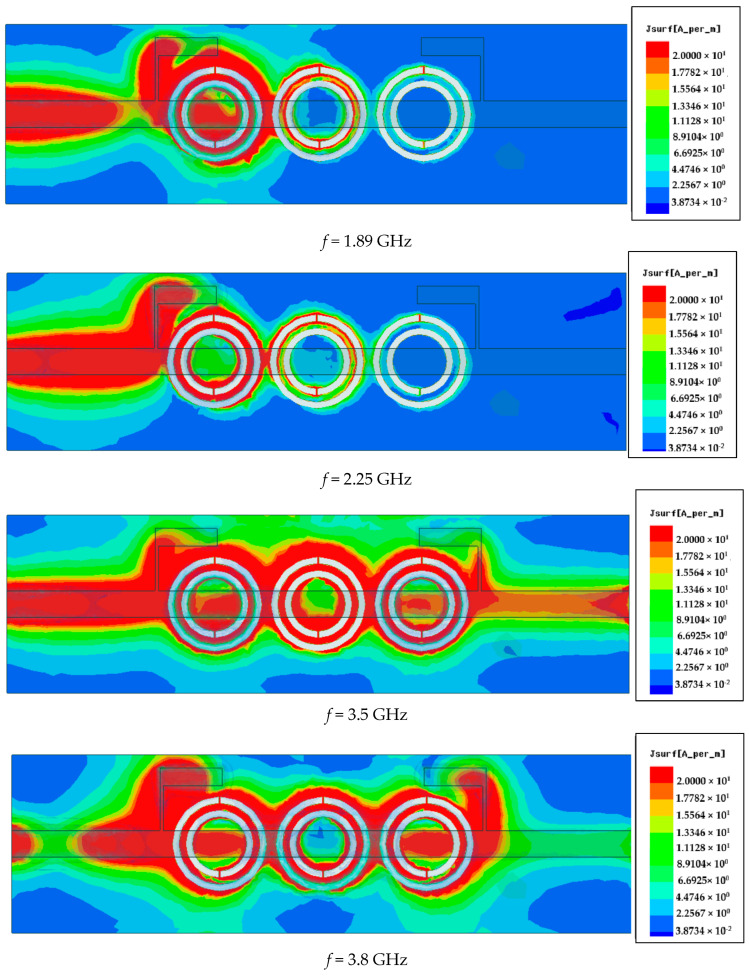
Simulated surface current profiles at lower cutoff (1.89 GHz), stopband center (2.25 GHz), upper cutoff (3.5 GHz), and out-of-band (3.8 GHz) frequencies.

**Figure 14:**
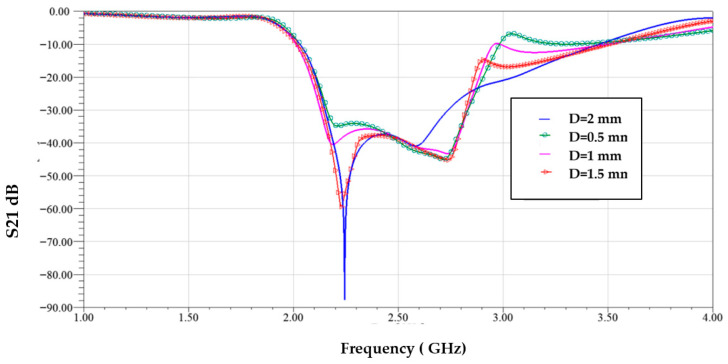
Transmission coefficient with different values of gap spacing between cells *D*.

Figure 13 illustrates the surface current distribution of the proposed resonator at four different frequencies: 1.89 GHz (lower cutoff), 2.00 GHz (within the stopband), 3.5 GHz (upper cutoff), and 3.8 GHz (out-of-band). At 1.89 GHz, the current is weak and concentrated near the input region, indicating minimal interaction with the resonator, which allows signal transmission—marking the edge of the passband. At 2.00 GHz, strong surface currents are observed circulating intensely around all three CSRR elements, indicating strong resonance and energy absorption. This confirms the frequency lies within the stopband, where the resonator effectively blocks transmission—exhibiting band stop filter (BSF) behavior. At 3.5 GHz, the current intensity starts to decrease and spreads more uniformly, indicating the transition back into the passband. At 3.8 GHz, very little current couples to the resonant structure, confirming this frequency is far outside the stopband and thus is fully transmitted. These results demonstrate that the resonator operates effectively as a band stop filter, strongly rejecting signals within a defined frequency range while allowing those outside the band to pass with minimal attenuation. Figure 14 shows the S21 transmission response for different values of the gap spacing D between the CSRR cells. Variations in D clearly affect the resonance depth and frequency behavior.

### 4.1. Inductive-Stub-Coupled CSRR Microwave Sensor for Non-Invasive Glucose Monitoring

**Figure 15:**
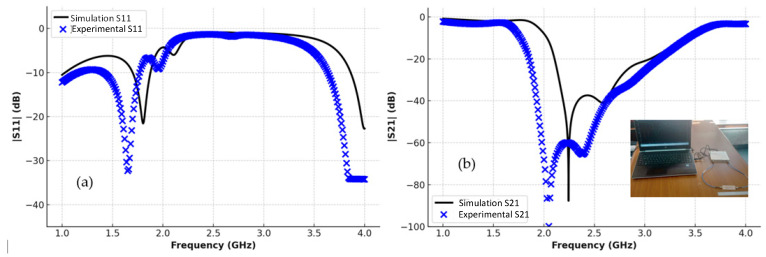
Experimental and simulated transmission and reflection responses of the CSRR sensor: (**a**) |S11|, (**b**) |S21|.

This section presents and explains the results obtained from the simulation of the designed sensor. Figure 15 shows the transmission coefficient versus frequency for two cases—the experimental and simulated phantom tissue, when D = 1.5 mm. As seen in Figure 15, the designed sensor exhibits a resonance at a center frequency of 2.4 GHz.

The experimental setup for the practical measurement is simple and compact. It consists of the designed CSRR sensor, connected directly to a Vector Network Analyzer (VNA) through a coaxial cable. The sensor is placed in a stable position on a flat surface, and the phantom or fingertip sample is positioned above the sensing area during measurement. The VNA is used to record the S21 transmission response over the selected frequency range. This configuration represents the practical environment for testing and verifying the sensor’s performance under realistic conditions.

The figure illustrates a comparison between the simulated (theoretical) and measured (practical) results for both the reflection coefficient (S11) and the transmission coefficient (S21). In both plots, the black curves represent the theoretical responses obtained from electromagnetic simulations, while the blue markers indicate the experimentally measured data. The results show a strong overall agreement in the resonance positions and trends, with slight variations in magnitude and bandwidth. These differences can be attributed to fabrication tolerances, measurement setup conditions, and losses not fully captured in the simulation model. The close correspondence between simulation and experiment confirms the validity of the proposed sensor design and demonstrates its reliable performance in practical implementation.

**Figure 16:**
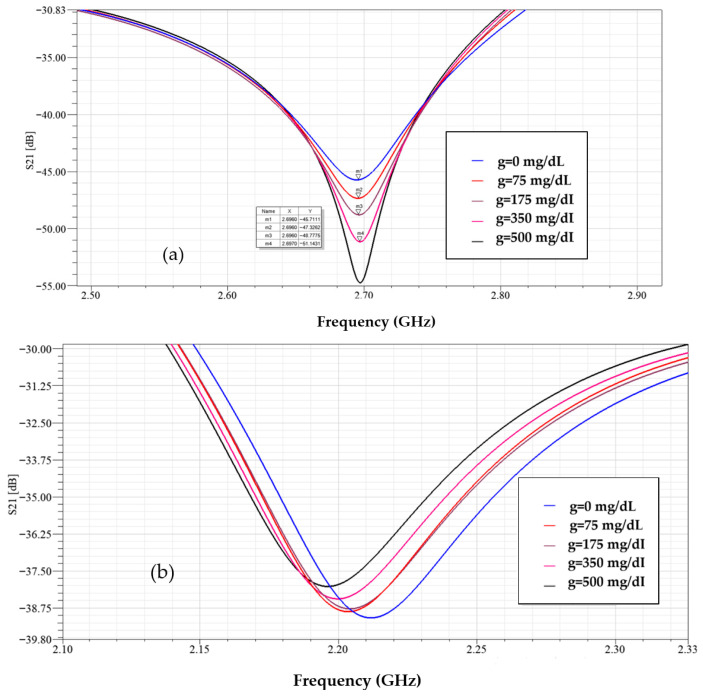
Simulated transmission response of the modified CSRR sensor loaded with different glucose concentrations at D = 1.5 mm, (**a**) *f* = 2.7 GHz, and (**b**) *f* = 2.2 GHz.

**Figure 17:**
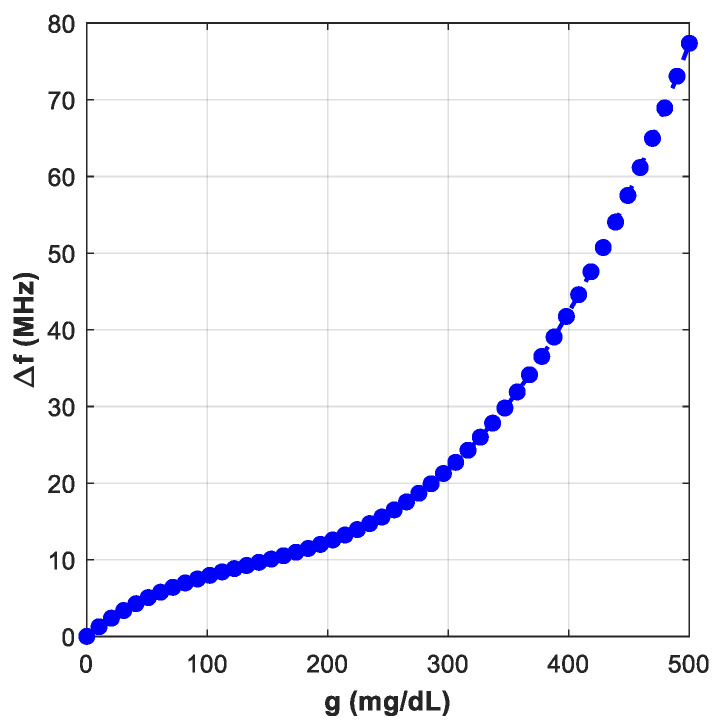
Variation of ∆*f* with g.

In Figure 16 with the sensor loaded by glucose phantoms (D = 1.5 mm), the transmission curves show two relevant resonances: (a) around 2.7 GHz, the resonance depth varies systematically with glucose—higher glucose produces a stronger attenuation at the notch and a small but consistent frequency drift, indicating a strong field–sample interaction; (b) around 2.2 GHz, the response also changes monotonically with glucose, but here the effect is observed as a combined notch-depth change and a measurable frequency shift. Together, panel (a) and (b) establish that both Δ|S21| and Δf are usable sensing observables. In Figure 17, from the spectra in Figure 16, the frequency shift Δf versus g is extracted. The curve is monotonic and increases with greater slope at higher g, reflecting the growing dielectric contrast. This is the basis for our reported frequency sensitivity of 0.086 MHz/(mg/dL).

**Figure 18:**
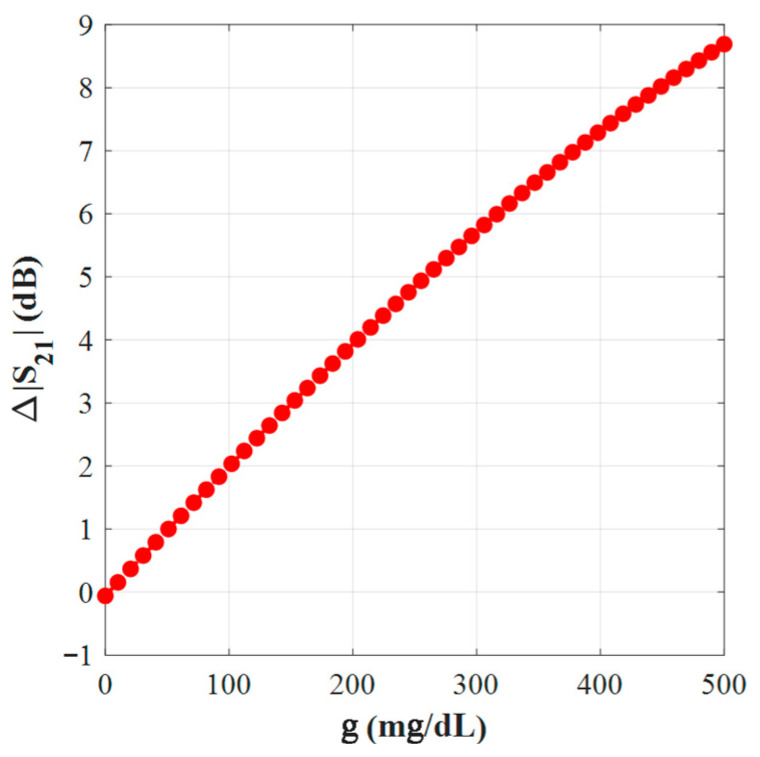
Variation of *∆S_21_* with g.

In Figure 18, similarly, the magnitude changes Δ|S21| (dB) grow almost linearly with g across the tested range, supporting robust calibration with a single-slope model. This corresponds to the measured amplitude sensitivity of 0.02 dB/(mg/dL).

**Figure 19:**
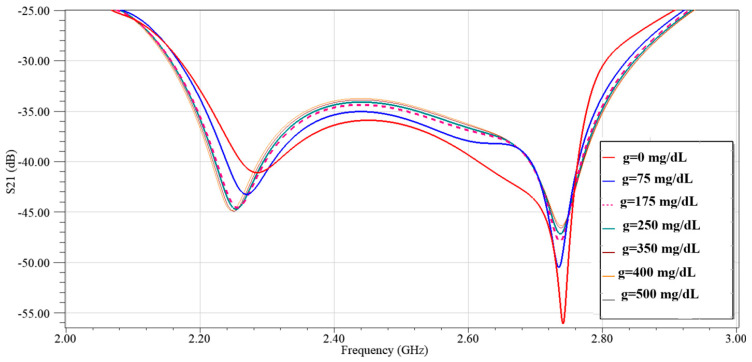
Simulated transmission response of the modified CSRR sensor loaded with different glucose concentrations at D = 2 mm.

In Figure 19 we repeated the analysis at a larger gap of D = 2 mm (same glucose set) which yields greater separation between curves and a sharper Q at both resonances (insets around ~2.23 GHz and ~2.7 GHz). The optimized gap reduces parasitic capacitive coupling, which improves the quality factor and enhances the sensing contrast.

**Figure 20:**
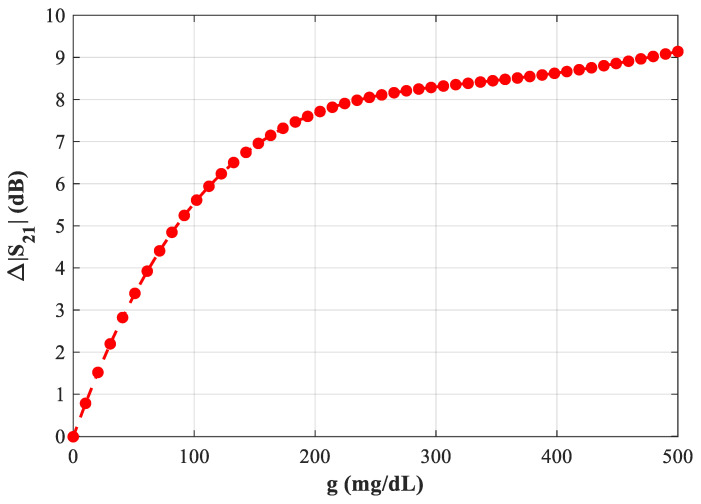
Variation of resonant frequency with increasing blood glucose levels in the proposed microwave sensor at *f* = 2.23 GHz.

Figure 20 summarizes the plot of Δ|S21| versus glucose at ~2.23 GHz (D = 2 mm), highlighting the practical operating region where the amplitude readout delivers a high signal-to-glucose ratio and good repeatability.

**Figure 21:**
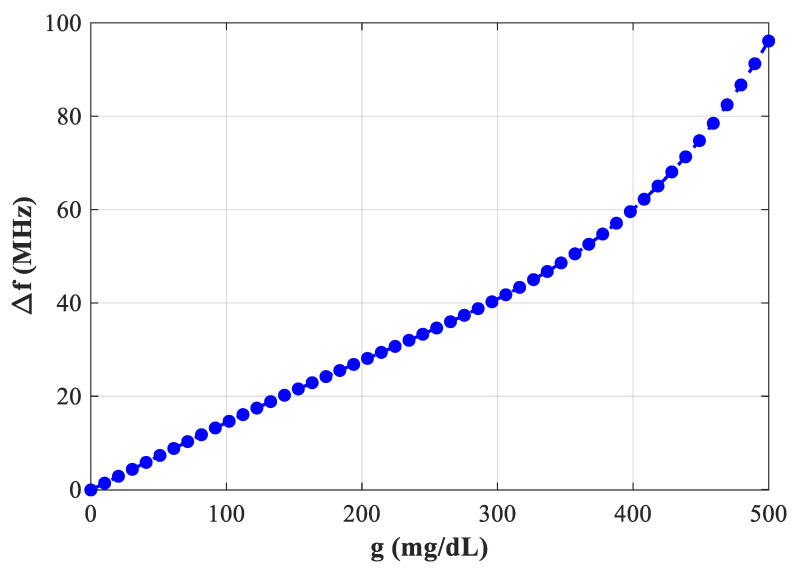
Variation of $\left|S21\right|$ increasing as a function of blood glucose concentration at f = 2.75 GHz.

Figure 21 represents the plot of Δf versus glucose at ~2.75 GHz (D = 2 mm). The trend remains strictly increasing, confirming that the frequency-based readout provides clear resolvable shifts over instrument uncertainty.

### 4.2. Sensitivity Analysis of Modified Inductive-Stub-Coupled CSRR Microwave Sensor

A comprehensive sensitivity analysis was conducted for the proposed Modified Inductive-Stub-Coupled CSRR Microwave Sensor, designed for glucose monitoring applications as shown in Table 5. The results reveal a clear enhancement in sensor performance due to the integration of inductive stubs, which effectively concentrate the electric field and amplify dielectric perturbations caused by the glucose variations.

The sensor was evaluated for two different stub gaps (D = 1.5 mm and D = 2 mm) across two resonance modes. For D = 2 mm, the sensor achieved its highest sensitivity, reaching 0.086 MHz/mg/dL in frequency shift and 0.02 dB/mg/dL in transmission coefficient magnitude variation at resonant frequencies of 2.3 GHz and 2.75 GHz, respectively. In comparison, the conventional CSRR sensor demonstrated significantly lower sensitivity, with values of 0.032 MHz/mg/dL and 0.0116 dB/mg/dL. This indicates a substantial improvement of approximately 168% in frequency-based sensitivity and 72% in magnitude-based sensitivity using the proposed design. These enhancements are attributed to the improved electromagnetic coupling and stronger resonance field distribution introduced by the stub-coupled architecture. Overall, the modified CSRR sensor exhibits superior performance, making it a promising candidate for high-resolution, non-invasive glucose sensing in biomedical applications.

The proposed modified CSRR sensor outperforms previous designs by achieving a high sensitivity of 0.086 MHz/mg/dL and 0.02 dB/mg/dL, which exceeds most of the values reported in the literature. Table 6 presents a comparison between the proposed non-invasive glucose sensing method and previously reported non-invasive measurement techniques from the literature, highlighting the achieved improvements in sensitivity and operating frequency. Compared to works [37,38,39,40], it shows at least 2–10 times improvement in detection resolution. Unlike invasive techniques [37], this sensor is fully non-invasive and compact. Operating at ~2.3 GHz, it remains compatible with wearable biomedical systems. These advantages highlight its potential for real-time, high-accuracy glucose monitoring.

## 5. Conclusions

This work presents the design, simulation, and sensitivity evaluation of a novel Modified Inductive-Stub-Coupled CSRR Microwave Sensor for non-invasive blood glucose monitoring. By integrating inductive stubs into the conventional CSRR architecture, the proposed sensor achieves superior field confinement and enhanced coupling efficiency, which significantly improves the sensitivity to dielectric variations in biological tissues, particularly blood glucose concentration.

Comprehensive simulations using a multilayer finger phantom model revealed that the modified sensor exhibits dual-resonance behavior, allowing glucose detection at multiple frequency bands. The sensor demonstrated maximum sensitivity at a stub gap of D = 2 mm, achieving 0.086 MHz/mg/dL for frequency shift and 0.02 dB/mg/dL for transmission coefficient variation. These results reflect a 168% improvement in frequency-based sensitivity and a 72% enhancement in amplitude-based sensitivity compared to the conventional CSRR sensor, which achieved only 0.032 MHz/mg/dL and 0.0116 dB/mg/dL, respectively.

Furthermore, the proposed sensor maintains a compact footprint, low cost, and compatibility with wearable or portable platforms, making it a promising candidate for real-time, continuous, and non-invasive glucose sensing in clinical and personal healthcare environments. Future research will focus on experimental validation, integration with wireless readout circuits, and further optimization for temperature compensation and individual-specific calibration.

## Figures and Tables

**Table 1:** Dimensions of CSRR based unit cell.

Cell Parameter	*L_s_*	*W_s_*	*R*_1_	*R*_2_	g	*h*	*w*
**Dimension (mm)**	30	20	5.34	4.47	7	1.6	0.76

**Table 2:** Unit cell extracted parameters.

Lumped Elements Value	*L_L_* (nH)	*C_L_* (pF)	*L_r_* (nH)	*C_r_* (pF)
**Dimension (mm)**	2.9095	1.125	1.752	1.95

**Table 3:** Proposed sensor dimensions.

Parameter	*L_s_*	*W_s_*	*R_1_*	*w*	X	*h*	*g*	*D*	*t*
**Dimension (mm)**	70	20	5.34	0.76	7	1.6	0.16	17	0.035

**Table 4:** Electrical and geometrical properties of human finger tissue layers used in phantom modeling.

Phantom Layer	*ε_r_*	*σ* (s/m)	*Thickness* (mm)
Skin	38–43	1.5–1.75	1.5
Fat	5.13–5.45	0.1–0.32	1
Blood	58–61	1.9–2.1	1
Muscle	48–52	1.6–1.7	2.5
Bone	9–10.8	1.65–1.75	3

**Table 5:** Sensitivity comparison of the modified CSRR sensor.

Stub Gap Dimension	*D* = 1.5 mm		*D* = 2 mm	
Frequency Band	1st f = 2.3 GHz	2nd 2.7 GHz	1st f = 2.3 GHz	2nd 2.75 GHz
**Sensitive Parameter**	$∆f\;\left(MHz\right)$	$\left|S21\right|\left(dB\right)$	$∆f\;\left(MHz\right)$	$\left|S21\right|\left(dB\right)$
***Sensitivity***	0.036 MHz/mg/dL	$0.018\frac{dB}{\frac{mg}{dL}}$	0.086 MHz/mg/dL	$0.02\frac{dB}{\frac{mg}{dL}}$

**Table 6:** Performance comparison of proposed antenna with the literature.

Reference	Technology	Operating Frequency (GHz)	Sensitivity
[37]	Active Split-Ring Resonator	1.1315	0.24 kHz/mMol/L ≈ 0.0043 MHz/mg/dL
[38]	Dielectric Resonator	4.7	0.002 MHz/mg/dL
[39]	Double split ring resonator	1.4	3.287 kHz per mmol/L ≈ 0.0655 MHz/mg/dL
[40]	Linear and Mediator-Free Resonator	1.5	0.0049 dB/mg/dL
[41]	CSRR resonator	2.95	0.0003 dB/mg/mL
[42]	Open-ended microstrip Transmission line loaded with CSRR	2.5	0.005 dB/mg/mL
[43]	Millimeter Waves using Microstrip Patch Antennas	60	0.65 × 10^−3^ dB/mg/dL
**This work**	Modified Inductive-Stub-Coupled CSRR	~2.3	0.086 MHz/mg/dL 0.02 dB/mg/dL

## Data Availability

The original contributions presented in this study are included in the article. Further inquiries can be directed to the corresponding author.
